# Advances in the pharmacological treatment of hepatic alveolar echinococcosis: From laboratory to clinic

**DOI:** 10.3389/fmicb.2022.953846

**Published:** 2022-08-08

**Authors:** Xiaolei Xu, Xinye Qian, Cancan Gao, Yuan Pang, Hu Zhou, Lizhen Zhu, Zhan Wang, Mingquan Pang, Defang Wu, Wenhao Yu, Fanyu Kong, Dalin Shi, Yuting Guo, Xiaoxia Su, Wang Hu, Jun Yan, Xiaobin Feng, Haining Fan

**Affiliations:** ^1^Department of Hepatobiliary and Pancreatic Surgery, Affiliated Hospital of Qinghai University, Xining, Qinghai, China; ^2^School of Clinical Medicine, Tsinghua University, Beijing, China; ^3^Qinghai Research Key Laboratory for Echinococcosis, Qinghai, China; ^4^Center of Hepatobiliary Pancreatic Disease, Beijing Tsinghua Changgung Hospital, Beijing, China; ^5^Department of General Medicine of Air Force Medical Center, Beijing, China; ^6^Department of Mechanical Engineering, Tsinghua University, Beijing, China; ^7^Biomanufacturing and Rapid Forming Technology Key Laboratory of Beijing, Beijing, China

**Keywords:** hepatic alveolar echinococcosis, drug repurposing, antibiotic drug, protease inhibitor, immune checkpoint inhibitor

## Abstract

Hepatic alveolar echinococcosis (HAE) is a zoonotic parasitic disease caused by the larvae of *Echinococcus multilocularis*. Because of its characteristics of diffuse infiltration and growth similar to tumors, the disability rate and mortality rate are high among patients. Although surgery (including hepatectomy, liver transplantation, and autologous liver transplantation) is the first choice for the treatment of hepatic alveolar echinococcosis in clinic, drug treatment still plays an important and irreplaceable role in patients with end-stage echinococcosis, including patients with multiple organ metastasis, patients with inferior vena cava invasion, or patients with surgical contraindications, etc. However, Albendazole is the only recommended clinical drug which could exhibit a parasitostatic rather than a parasitocidal effect. Novel drugs are needed but few investment was made in the field because the rarity of the cases. Drug repurposing might be a solution. In this review, FDA-approved drugs that have a potential curative effect on hepatic alveolar echinococcosis in animal models are summarized. Further, nano drug delivery systems boosting the therapeutic effect on hepatic alveolar echinococcosis are also reviewed. Taken together, these might contribute to the development of novel strategy for advanced hepatic alveolar echinococcosis.

## Introduction

Hepatic echinococcosis (HE) is a zoonotic parasitic disease that seriously endangers people’s health and socio-economic development worldwide, especially in some rural area of China (McManus et al., 2003; Yang et al., 2017). The annual treatment costs and livestock losses associated with the disease are expected to be $3 billion (Kern et al., 2017), which has become a major global public health problem. In 2010, the World Health Organization listed it as one of the 17 neglected diseases that should be controlled or eliminated by 2050. 91% of the new cases in the world come from China each year (Qian et al., 2017).

There are mainly two kinds of echinococcosis: hepatic cystic echinococcosis (HCE) and hepatic alveolar echinococcosis (HAE). HE is caused by the parasite passing through the intestinal mucosa and then entering in the liver through the portal vein system. HAE only accounts for 5–10% of the overall incidence rate of echinococcosis (Akbulut et al., 2014; Akbulut, 2018). Although it is a benign disease, its growth mode is like a malignant disease (Parsak et al., 2007). HAE could not only infiltrate into surrounding tissues such as gallbladder, blood vessels, or biliary system, but also distal organs, such as kidney, lung, brain, bone, etc. (Hatipoglu et al., 2013; Joliat et al., 2015). Although surgery is the ideal treatment for HAE, patients are constantly diagnosed at the advanced stage of the disease as the initial symptoms can be sub-clinical. Also, lack of health knowledge and medical resources makes the problem worse. As a result, many HAE patients lose the opportunity for surgery, leaving medication the only option for these patients.

Mebendazole and Albendazole are mainly used to treat advanced HAE. Study (Davis et al., 1989; El-On, 2003) showed that albendazole is more efficient than mebendazole in the treatment of HAE. Albendazole can inhibit the uptake of glucose, deplete the glycogen of the germinal layer cells, denature the endoplasmic reticulum bodies and mitochondria, increase the lysosomes, and finally lead to the death of the parasite. However, albendazole exhibit a parasitostatic rather than a parasitocidal effect. The plasma concentration of albendazole was only (1.86 ± 0.88) mg/L, while the drug concentration in hydatid lesions was only 1/100 of the plasma concentration, which was far lower than the concentration required to kill multilocular *Echinococcus multilocularis*. Further, advanced HAE cases must undergo long-term chemotherapy (often life-long), which could result in adverse complications such as abnormal liver function, leukopenia, and hair loss (Meilinger et al., 2013). Therefore, recurrence rates after interruption of therapy are relatively high (Reuter et al., 2004). In addition, long-term drug use is easy to cause drug resistance of parasites (Pawluk et al., 2015), also resulting in the progression of the disease. As a result, effective novel drugs are needed for the disease.

## Dilemma

For many years, the pharmaceutical industry has not been compelled to develop novel drugs against HAE due to its relatively rare abundance, investments in the development of new drugs against AE will not result in a high market return, resulting in Low investment of the area. Also, the rarity of the cases would make it almost impossible to conduct clinical trials like randomized control trial, or cohort study, etc. So, even there is a new drug for HAE, it would take years to investigate its efficiency and safety in HAE patients. Up to date, Mebendazole and Albendazole are still the only options for these patients.

Drug repurposing or studying existing drugs for potential therapeutic utility in newer indications has been identified as an attractive option for treating a number of diseases (Polamreddy and Gattu, 2019), which might be a solution for this problem. Drug repurposing offers manifold advantages over *de novo* drug discovery and development process such as expedited and economical drug development process (Breckenridge and Jacob, 2019), curtailing the development risks as safety of the compound, a major reason for high attrition rates in drug development process, is already well established (Gupta et al., 2013). Due to huge promise offered by drug repurposing compared to conventional drug development process, this strategy is being adopted by many pharmaceutical companies to redevelop their approved and shelved molecules as novel therapeutic options in a wide range of indications (Neuberger and Oraiopoulos, 2019). Four pillars were identified to assure the success rate in drug repurposing programs (Mittal and Mittal, 2021). Pillar 1: drug pharmacology – sound knowledge of the repurposed drug’s pharmacological characteristics; pillar 2: drug formulation – drug formulation considerations in new indication; pillar 3: evaluation in biological assays – evaluation in representative biological assays with translational potential; pillar 4: clinical evaluation – robust clinical trial methodologies including biomarker-driven approach to provide conclusive evidence of repurposed drug’s efficacy in new indication.

Excitingly, lab experiments have showed that many FDA-approved drugs might have therapeutic effects on HAE. Many of the studies have provided Pillar 1, 2, or 3 evidence for drug repurposing, which might bring new opportunity for advanced HAE patients. In addition, these drugs could be used with Albendazole simultaneously in HAE patients. With these evidence, clinical trials could be enrolling advanced HAE patients for experimental treatment to improve outcome of these patients. In this review, the details of these potential drugs are being reviewed and summarized.

## Recent advances in drugs from lab

### Antibiotic drugs

Mefloquine, the famous antimalarial drug (Lundström-Stadelmann et al., 2020), showed a significant effect on reduction in parasite growth in mice infected with multilocular Echinococcus intraperitoneally compared with the standard treatment with albendazole (to assess the viability of the parasite, mefloquine-treated metacestodes from *in vitro* cultures were injected into Balb/c mice. No parasite growth was observed after 5 months of incubation), although its exact role as a drug target remains to be clarified. Consider that HAE is not only a parasitic disease, but also a infectious disease. It is reported that (Mai et al., 2011) broad-spectrum anti-infective drug - nitazonit (nitazoxanide) also showed its effect in killing the parasites. *In vitro* experiment, no regrowth of cysts was observed even after 3 weeks, 3 months, or 6 months of nitazoxanide and albendazole administration. *In vivo* test, no metacestodes of the hydatid were found in mice treated by nitazoxanide. In addition, amphotericin B has been proved to effectively inhibit the growth of Echinococcus by using the *in vitro* culture model. When amphotericin B was added to metacestode tissue after 10 weeks of culture, destruction of vesicles was observed as soon as 1 day after the start of treatment, and all vesicles were disrupted after 8 days (Reuter et al., 2003). The antifungal drug amphotericin B has been applied to patients as a remedial treatment, but it is not an insecticidal drug, and long-term use will cause nephrotoxicity.

### Protease inhibitor

Hepatic alveolar echinococcosis, similar to tumors, proliferates by budding or infiltrating into tissues, and constantly promoting angiogenesis. It has been suggested that the unlimited proliferation ability of Echinococcus may be related to some signal transduction protein kinase molecules (Siles-Lucas et al., 2001). As a phosphoserine/phosphothreonine binding module, 14-3-3 protein is involved in the process of DNA damage checkpoint and prevention of apoptosis (Wilker and Yaffe, 2004). In fact, the 14-3-3 cDNA sequence of Echinococcus was compared with the 14-3-3 isomer of other organisms, and the parasite sequence was classified as the isomer related to tumor growth (Spiliotis et al., 2005). Britta Stadelmann et al. screened a library containing 426 FDA-approved drugs (Stadelmann et al., 2014). The study showed that bortezomib (BTZ), a proteasome inhibitor, could cause parasite death *in vitro* model. The animal model also showed that the parasite weight was reduced by an average of 2 g under a BTZ dosage of 0.5 mg/kg combined with ABZ, proving that bortezomib can be used as a drug target in multilocular Echinococcus. Moreover, GTPases Ras and RAF, and genes encoding epidermal growth factor receptors, were found in *Echinococcus Taenia* (Spiliotis et al., 2003). RhoA, a protein with GTPase activity in Ras superfamily, activates target proteins by binding to GTP. RhoA could activate RAF, which is an upstream protein of MAPK signaling pathway (Kontaridis et al., 2008). Activated Raf further activates MEK, which is responsible for ERK and JNK phosphorylation (Ke et al., 2019). Activated ERK and JNK induce macrophage polarization by activating certain transcription factors (Weiss et al., 2018). MAPK signaling pathway is crucial for parasitic infection (Zhao et al., 2019). Shigui Chong et al. found that soluble antigen of *Echinococcus multilocularis* induces macrophage polarization after alveolar echinococcosis infection through RhoA MAPK signal pathway (Chong et al., 2022). Emmpk1, a host extracellular signal regulated kinase (ERK), could activate parasitic MAPK cascade as it could be phosphorylated by multilocular Echinococcus cysts *in vitro* (Hemphill et al., 2002; Spiliotis et al., 2006). MAPK signaling pathways, including c-Jun N-terminal kinase (JNK), p38 MAPK and ERK, play an important role in signal transduction from cell membrane to nuclear transcription factors, balancing cell survival and death in liver injury (Ballif and Blenis, 2001). Ren Yong Lin et al. reported multilocular echinococcosis could directly affect hepatocyte proliferation *via* MAPK pathway (Lin et al., 2009). The team also reported that MAPK signaling pathway was upregulated by microarray analysis in mice infected with *Echinococcus multilocularis* (Lin et al., 2011). According to these findings, MAPK pathway could be a therapeutic target for HAE.

MAPK pathway inhibitors have been applied in clinic for decades. Sorafenib is a small molecule anticancer drug targeting RAF in Raf/ERK signaling pathway to inhibit tumor cell proliferation and angiogenesis by targeting tyrosine kinases, such as intravascular growth factor receptors VEGFR-2 and VEGFR-3 and platelet-derived growth factor receptor (PDGFR). Xiao explored the efficacy of different concentrations of anti-cancer drug sorafenib in multilocular echinococcosis through animal models. The results showed that sorafenib showed strong cyst inhibition effect and was a potential drug for the treatment of echinococcosis (the cyst inhibitory rate of sorafenib was 6.6, 42.4, 68.5, 77.4, 84.0 and 89.5% at 10 μmol/l, while the cyst inhibitory rate of albendazole was 3.8, 12.7, 27.0, 51.4, 54.0, and 73.0% at 30 μmol/l after 48, 72, 96, 120, 144, and 168 h of administration; Xiao, 2021).

Hemer and Brehm also focused on parasite signaling pathways when looking for new drug targets. It was found that imatinib, a TKI tyrosine kinase inhibitor, has the ability to efficiently kill the protoscolex of Echinococcus *in vitro*. In addition, imatinib could significantly inhibit the formation of prototapeworm vesicles by parasitic stem cells at concentrations as low as 10 m. After 7 days of treatment, 50% of the vesicles lose activity and induce morphological changes of tapeworm. Therefore, imatinib might become a promising alternative to albendazole in anti-echinococcosis chemotherapy (Hemer and Brehm, 2012). Although little is known about the signal transduction pathway, growth factors and tyrosine kinase signaling molecules might play a vital role in the process of worm differentiation and reproductive activity, which might be potential targets for new drug treatment of echinococcosis. Recent progress in the characterization of TKI and signals confirms the importance of tyrosine kinase activation pathway in larvae (Brehm et al., 2006). Based on these facts, TKI inhibitors could be potential strategies for treating echinococcosis (Dissous et al., 2007).

### Immune checkpoint inhibitor

Echinococcus can parasitize in the host for a long time without being cleared by the body’s immune system is because the antigen produced by Echinococcus after entering the host can regulate the body’s immune microenvironment through a variety of ways (Zhang et al., 2016), including escaping the attack of host immune cells, and inducing the immunosuppression environment. It is found that the immune microenvironment is of great significance in the occurrence and development of a variety of diseases, and the change of immune environment also provides a suitable environment for the survival and reproduction of multilocular Echinococcus (Bakhtiar et al., 2020). Multiple evidence show that the hepatic inflammatory microenvironment is crucial in the development of hepatic echinococcosis (Gottstein et al., 2017).

The early stage of animal infection is characterized by Th1/Th2 mixed immune response, which is accompanied with an increase in interferon-γ (IFN-γ), interleukin-4 (IL-4), and related chemokines. In human HAE, CD4+/CD25+ regulatory cells (Tregs) play a major role in regulating immune response and seem to be upregulated in the time course of the disease, which might be related to the suppression of the immune response to specific antigens and promotion of the secretion of anti-inflammatory cytokines like interleukin-10 (IL-10), transforming growth factor β (TGF- β), etc. (Vuitton et al., 2006). Studies showed that prolonged exposure to IL-10 could lead to T cell dysfunction (Monaghan et al., 2012). Similarly, TGF-β also could reduce the intensity of T cell response and induce T cell apoptosis, which is related to PD-1 signaling pathway (Ma et al., 2013). Xiaolin La et al. explored the relationship between PD-1 / PD-L1 pathway and Tregs in different stages of multilocular Echinococcus infection. The expression of PD-1 and PD-L1 increased with the development of HAE erosion. The results also indicated that high expression of PD-1/PD-L1 might play an important role in stimulating CD4+/CD25+ T cells, maintaining peripheral tolerance and immune escape during chronic infection of Echinococcus (La et al., 2015). Junhua Wang et al. evaluated the effect of regulatory T cell (Treg) deficiency on the growth of *Taenia saginata*. After Treg deficiency was induced by diphtheria toxin (DT), the parasitic lesions (>4 mm) in the liver of mice infected with multilocular bacteria were significantly smaller than those (<2 mm) in the corresponding control group (Wang et al., 2018).

Anne Pauline Bellanger et al. analyzed the cells and serum of 22 healthy blood donors. After blood samples were stimulated with multilocular Echinococcus, a significant increase in PD-L1 and CTLA-4 was observed (Bellanger et al., 2020), suggesting that PD-L1 pathway plays an important role in the infection of multilocular *Echinococcus multilocularis* (Wang et al., 2018). Fadi jebbawi et al. started immunotherapy 6 weeks after multilocular *E. coli* infection and maintained it for 8 weeks (Jebbawi et al., 2021). Mice were treated with albendazole (orally for 5 days / week) and PD-L1 (twice a week intraperitoneally). The results showed that PD-L1 blocker can significantly reduce the size and weight of parasitic lesions by increasing CD4/CD8 effector T cells and reducing Treg, which indicates that PD-1/PD-L1 pathway blocking has great potential in the treatment and control of multilocular Echinococcus infection. Chuanshan Zhang et al. found that TIGIT might also be a potential therapeutic target for HAE as liver weight, lesion weight, lesion area, and confluent lesion numbers were significantly lower in TIGIT blocked mice as compared with control mice (Zhang et al., 2020). Natural killer (NK) cells are active members of innate immunity. More and more studies have proved their importance in liver immunity (Kubes and Jenne, 2018). Although the mechanism of NK cell in HAE is not fully understood, Hao Wen and colleagues explored TIGIT as an important potential biomarker in HAE patients and *E. multilocular-infected* mice. The lack or blocking of TIGIT partially inhibits the growth of E. multilocular by reversing the damaged function of NK cells. The results suggest that targeting TIGIT may be a potential immunotherapeutic strategy for the treatment of patients with AE (Zhang et al., 2021; Table 1).

**Table 1:** Recent advances in drugs for hepatic echinococcosis.

Country or territory	Drug type	*In vivo*	*In vitro*	Anticancer ingredient	References
Switzerland	Antibiotic		√	Mefloquine	Lundström-Stadelmann et al. (2020)
China	Antibiotic	√	√	Nitazoxanide	Mai et al. (2011)
Germany	Antibiotic		√	Amphotericin B	Reuter et al. (2003)
Switzerland	Proteasome inhibitor	√		BTZ	Stadelmann et al. (2014)
China	Multikinase inhibitor		√	Sorafenib	Xiao (2021)
Germany	PTKI		√	Imatinib	Hemer and Brehm (2012)
Switzerland	PD-1 inhibitor	√	√	PD-1 inhibitor	Jebbawi et al. (2021)
China	TIGIT inhibitor	√	√	TIGIT inhibitor	Zhang et al. (2021)

BTZ, Bortezomib. PTKI, Protein tyrosine kinase inhibitor.

### Chinese herbal medicine

Previous studies have reported the efficacy of Chinese herbal medicine in the treatment of echinococcosis. Liu et al. (2021) have tested the anthelmintic effect of saffron on the pathogenic scolex of Echinococcus both *in vitro* and *in vivo*. They found that saffron inhibits the expression of matrix metalloproteinases (MMPs), particularly MMP2 and MMP9, in the host tissue around the tapeworm, which could promote the deposition of collagen in the protoscolex. The results showed that saffron could be developed as a new drug for the treatment of HAE.

Artemisinins have been found to have antiparasitic effect *via* various mechanisms, such as ROS-dependent depolarization of the membrane (Antoine et al., 2014), or reactive oxygen species mediated DNA damage (Gopalakrishnan and Kumar, 2015), etc. Artesunate is a semi-synthetic derivative of artemisinin. Its water solubility determines its good absorption, high bioavailability, and long half-life *in vivo*. Meanwhile, it could be prepared into oral, rectal, intramuscular, and intravenous dosage forms, which is convenient for clinical administration. Xiao et al. (2008) proved the inhibitory effect of artesunate on adult *Clonorchis sinensis in vivo* through a rat model as significant worm burden reductions was observed after artesunate was administrated. Jiraungkoorskul et al. (Jiraungkoorskul et al., 2005) observed, *in vitro*, that the surface layer of the artesunate-treated *Schistosoma japonicum* was seriously damaged and its activity decreased. These findings suggested that artemisinins might have an important role in treating HAE through further validation would be needed.

Jiang C et al. have created a self-made pure traditional Chinese medicine “Xiaobao Decoction” to treat hydatidosis in mice. The inhibition rate of alveolar hydatidosis was 65.7% ~ 80.6% (Jiang, 1995), indicating that the traditional Chinese medicine Xiaobao Decoction might be a promising alternative or adjuvant therapy for HAE (Jiang, 1998). The main ingredients of Xiaobao Decoction are areca, Begonia, snake slough, ground beetle, honeycomb, and scales. In order to achieve better anti-echinococcosis effect, various dosage forms of Xiaobao Decoction were also produced, such as Xiaobao capsule, Xiaobao tablet, etc. Jiang CP et al. further explored the therapeutic effect of Xiaobao pill combined with albendazole in the treatment of hydatidosis, founding that the combination caused more serious damage to the ultrastructure of hepatic multilocular echinococcosis than albendazole alone (Jiang, 1991). Clinical data also showed that Xiaobao pill has a good therapeutic effect on hydatidosis (Jiang, 1986; Jiang and Liu, 1994). However, multi-center clinical study with large sample size is still needed to evaluate its antiparasitic effect.

### Nano drug delivery system

Although the traditional tablet albendazole (t-abz) has curative effect on hydatid, its clinical cure rate is low. The reason for this is that ABZ is poorly and erratically absorbed following oral administration resulting in low drug levels in plasma and liver distribution. Therefore, there is an urgent need for designing new formulations of ABZ with increased bioavailability to improve the pharmacokinetic effects of drugs. Liposomal chemotherapy are applied in clinics as they could gather in the targeted tissues and release the drug continuously (Yue and Dai, 2018), which could solve the limitation of traditional tablet albendazole. Thus, drug repositioning combined with nanotechnology to improve drug bioavailability becomes a useful, fast, and inexpensive tool for the treatment of neglected diseases. To elevate the low water solubility, poor absorption, and low bioavailability of Albendazole, Rodrigues et al. (1995) established poly (D, L-lactide) nanoparticles loading with ABZ to investigate this new drug delivery system against *E. multilocularis* using a mouse model of hepatic alveolar echinococcosis. These treatments significantly reduced parasite node surface size as well as peritoneal metastatic burden compared to untreated mice as the concentration of drug was higher at the focus of infection compared with that of the controlled group. Nayer Mehdizad Bakhtiar et al. also showed that ABZ-loaded polymeric nanoparticles (NPs) had a tendency to increase the mortality rate against protoscoleces and microcysts compared with traditional albendazole (Bakhtiar et al., 2019). Pensel et al. suggest that ABZ nanocrystals (ABZ-NCs) seem to be a useful tool to increase bioavailability (Pensel et al., 2018). The preventive efficacy of the ABZ-NC preparation in mice infected with *Echinococcus multilocularis* was studied, and it was found that the mean weight of vesicles recovered in the ABZ-NC group was 50% lower than in the untreated mice. The survival rate of protospores isolated from ABZ-NC-treated mice was significantly lower than that of the control group (*p* < 0.05). After treatment with ABZ-NCs, the cyst weight was reduced by 77%, and the survival rate of its pronuclei was reduced to 34%, which is due to the increased oral bioavailability. Chunhui Hu et al. established a novel nanocrystalline (NC) formulation of ABZ by spray drying ABZ with a triblock copolymer poly(ethylene oxide)-poly(propylene oxide)-poly(ethylene oxide) (Poloxamer 188), and its physical structure was confirmed by scanning electron microscopy (SEM; Hu et al., 2020). The significant reduction in ABZ crystallite size coupled with the prolonged ABZ supersaturation greatly improved the dissolution properties of the drug compared to the commercial ABZ oral product (Albenda), and in a pharmacokinetic comparison, the NC formulation showed an approximately 4.2-fold higher AUC than Albenda, as measured by the plasma concentration of the active antiparasitic metabolite albendazole. More encouragingly, after 30 days of oral administration of NC and Albenda formulations once a day in SD rats with hepatic alveolar echinococcosis, the NC formulation exhibited 3.7 times more vesicle-inhibitory effect than Albenda. Therefore, NC formulations have the potential to be developed as an improved anti-AE drug therapy (Table 2).

**Table 2:** Nano drug delivery system of Albendazole.

Administrative region	*In vivo*	*In vitro*	CE	AE	Drug delivery system	References
France	√			√	ABZ-NPs	Rodrigues et al. (1995)
Iran and Italy	√		√	√	BMZ-NPs	Bakhtiar et al. (2019)
Argentina	√	√		√	ABZ-NCs	Pensel et al. (2018)
China	√			√	NC-ABZ	Hu et al. (2020)
China	√		√		ABZ-CS-NPs	Liu et al. (2013)
Iran		√	√		ABZ-NCs	Fateh et al. (2021)
Iran and United Kingdom		√	√		NLCs-IVM	Ahmadpour et al. (2019)
Iran	√	√	√		ABZ-SLNs	Aminpour et al. (2019)

CE, Cystic echinococcosis; AE, Alveolar echinococcosis; ABZ-SLNs, Albendazole loaded Solid lipid nanoparticles (SLNs); NLCs-IVM, Nano lipid carriers (NLCs)-loaded ivermectin (IVM); ABZ-NCs, Albendazole nanocrystals; ABZ-CS-NPs, Albendazole-associated chitosan nanoparticles; BMZ-NPs, BMZ-loaded polymeric NPs; ABZ-NCs, ABZ nanocrystals; NC-ABZ, Nanocrystalline formulation of ABZ.

As nano vehicle could increase local drug concentration and bioavailability, other anti-HE might also be loaded in these vehicles to achieve better therapeutic effects. Recently, single-dose liposomal amphotericin B combined with flucytosine and fluconazole was proved to be noninferior to the WHO-recommended treatment for HIV-associated cryptococcal meningitis and was associated with fewer adverse events (Jarvis et al., 2022), showing its merit in regional drug concentration and persistence. As amphotericin B was shown to be effective in inhibiting the growth of Echinococcus, and the antifungal drug amphotericin B was applied to patients as a remedial treatment. The liposomal amphotericin B might be a potential drug for HAE although it needs further validation.

## Summary

Albendazole is the only recommended clinical drug for advanced hepatic alveolar echinococcosis. However, its therapeutic efficiency is not optimal. It is reported that antibiotic (Mefloquine, nitazoxanide, and amphotericin B), protease inhibitor (bortezomib, Sorafenib, and imatinib), and immune checkpoint inhibitor (anti-PD-1/PD-L1 drugs, anti-TIGIT drugs) have anti-echinococcosis effect in animal models. Clinical trails investigating these drugs could be considered to achieve a parasitocidal effect rather than a parasitostatic effect. Moreover, nanoparticles loading Albendazole or other potential drugs could increase local drug concentration to treat echinococcosis efficiently. As there are already many nanoparticle could be applied in clinics, such as liposome, it could also be a strategy to develop novel drugs for HAE. Drug repurposing and nano drug delivery system might be promising in novel treatment for advanced alveolar echinococcosis.

## Author contributions

XX, XQ, and CG wrote the manuscript. YP, HZ and LZ designed form. ZW, MP, and DW checked out antibiotic drugs. WY, FK, and DS checked out protease inhibitor. YG, XS, and WH checked out immune checkpoint inhibitor. JY checked out immune checkpoint nano drug delivery system. XF and HF reviewed the manuscript. All authors contributed to the article and approved the submitted version.

## Funding

This study was supported by the Qinghai Provincial Science and Technology Department Project (No. 2020-ZJ-Y01), Young and Middle-aged Scientific Research Fund Project of the Affiliated Hospital of Qinghai University (No. ASRF-2021-YB-17), and Beijing Natural Science Foundation (Z190024).

## Conflict of interest

The authors declare that the research was conducted in the absence of any commercial or financial relationships that could be construed as a potential conflict of interest.

## Publisher’s note

All claims expressed in this article are solely those of the authors and do not necessarily represent those of their affiliated organizations, or those of the publisher, the editors and the reviewers. Any product that may be evaluated in this article, or claim that may be made by its manufacturer, is not guaranteed or endorsed by the publisher.
